# Ketogenic Diet Promotes Reward Learning by Upregulating Hippocampal CAMK2A Expression and Activating Dopamine Synaptic Signaling

**DOI:** 10.3390/ijms27083587

**Published:** 2026-04-17

**Authors:** Yanan Qiao, Yubing Zeng, Chen Chen, Jinying Shen, Yi Wang, Pei Pei, Shan Wang

**Affiliations:** 1 Capital Center for Children’s Health, Capital Medical University, Capital Institute of Pediatrics, Beijing 100020, China; qiaoyanan5114@126.com (Y.Q.); awater007@sina.com (Y.Z.); chencoeur@163.com (C.C.); 13726256164@163.com (J.S.); wang_yii@163.com (Y.W.); 13661202592@163.com (P.P.); 2 Capital Institute of Pediatrics, Chinese Academy of Medical Sciences & Peking Union Medical College, Beijing 100020, China

**Keywords:** ketogenic diet, dopaminergic synapse, reward learning, CAMK2A

## Abstract

Various neuromodulatory benefits of the ketogenic diet (KD) have been demonstrated, yet its influence on reward learning and underlying mechanisms remain poorly defined. This study combined proteomics and metabolomics to identify key molecular changes in the hippocampus of KD-fed mice. Our analysis revealed significant upregulation of the “dopaminergic synapse” pathway, with CAMK2A emerging as a central regulator. In vitro, treatment of the hippocampal neuronal cell line HT22 with β-hydroxybutyrate (BHB), a primary KD metabolite, increased the protein expression of CAMK2A and increased the phosphorylation of its downstream target, GluA1. Crucially, *Camk2a* knockdown completely blocked BHB-induced p-GluA1 enhancement. To determine the behavioral relevance, we stereotaxically delivered AAV-sh*Camk2a* into the hippocampus of KD-fed mice. Knockdown of *Camk2a* reversed the pro-reward effects of KD, as measured by the sucrose preference test and conditioned place preference test, without impairing general locomotor activity in the open field test. Together, these results suggest a novel BHB–CAMK2A–dopaminergic signaling axis through which KD enhances reward learning, thus bridging systemic metabolism with cognitive function and expanding our understanding of KD-mediated neuromodulation.

## 1. Introduction

Reward learning is central to motivated behavior and adaptive decision-making. Its neurobiological basis primarily involves the dopaminergic signaling pathways of the mesolimbic system [1,2]. While the dopamine projection from the ventral tegmental area (VTA) to the nucleus accumbens (NAc) has traditionally been considered the “central hub” of reward processing [3,4], growing evidence indicates that the hippocampus, as a key brain region for learning and memory [5], also considerably participates in the encoding and integration of reward information [6,7]. Moreover, the hippocampus not only receives dopaminergic inputs from the midbrain but also possesses its own independent and complex dopamine signaling system [8]. This local system can directly modulate synaptic plasticity to influence the formation of reward-related memories [9]. Investigating the regulatory mechanisms of dopamine signaling within the hippocampus is therefore crucial for a comprehensive understanding of the brain-wide network mechanisms underlying reward learning.

A ketogenic diet (KD), a dietary regimen characterized by high fat and very low carbohydrate intake [10,11], was first clinically applied in the 1920s for the treatment of refractory epilepsy [12,13,14]. In recent years, as its broad neuroprotective effects have been progressively revealed, research on the KD has expanded beyond anticonvulsant therapy to various domains [15]. Substantial evidence has demonstrated that the KD can improve cognitive performance [16], enhance spatial learning and memory [17], and influence reward-related behaviors. However, how the KD enhances reward learning ability, especially its regulatory effect on the signaling network within the hippocampus, has not been fully elucidated.

A hallmark metabolic feature of the KD is the hepatic production of ketone bodies [18,19], among which β-hydroxybutyrate (BHB) is the most abundant in circulation [20]. Historically regarded merely as an alternative energy source, BHB is now recognized as an important signaling molecule [21,22]. It has various epigenetic and signaling regulatory functions, including the inhibition of histone deacetylases [23,24,25] and the activation of specific G protein-coupled receptors [26]. This paradigm shift suggests that the neurological benefits of a KD may stem not only from a shift in energy metabolism but also from key signal transduction events triggered by BHB. CAMK2A, one of the most abundant protein kinases in the central nervous system [27,28], is a critical regulator of glutamate receptor function and synaptic plasticity [29]. It also plays a pivotal role in cross-talk with dopaminergic signaling pathways [30,31]. However, whether and how KD modulates hippocampal dopamine signaling and reward learning via BHB-mediated regulation of CAMK2A remain unknown.

On the basis of this background, we hypothesized that a KD enhances reward learning by upregulating hippocampal CAMK2A expression via its key metabolite BHB, thus activating local dopaminergic synaptic signaling. To test this hypothesis, we integrated proteomics, metabolomics, behavioral assays, molecular biology, and viral-mediated gene manipulation. Unbiased omics profiling first revealed the dopaminergic synapse pathway and its core component CAMK2A in the hippocampus under KD conditions. We then validated the BHB–CAMK2A signaling axis and its downstream mechanisms in hippocampal neurons. Finally, hippocampal-specific knockdown of *Camk2a* in vivo established its necessity for KD-enhanced reward behavior. This multilevel investigation, from systems and cellular analyses to in vivo behavior, revealed a novel mechanism through which KD regulates cognitive function and provides a new theoretical link between metabolic intervention and improved brain function.

## 2. Results

### 2.1. Ketogenic Diet Enhanced Reward Perception in Mice

**Figure 1:**
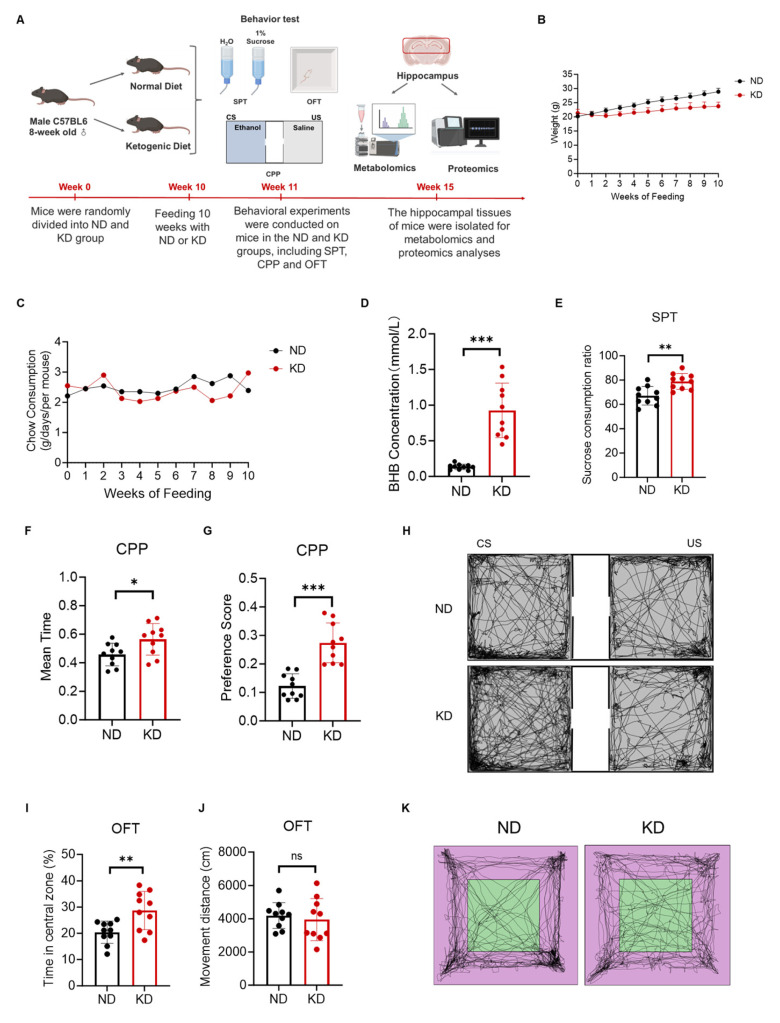
Ketogenic diet enhanced reward learning in mice. (**A**) Schematic diagram of the experimental timeline for KD intervention and behavioral tests. (**B**) Body weight of mice fed KD or ND (*n* = 10 per group). (**C**) Average daily food intake of mice fed KD or ND (*n* = 10 per group). (**D**) Serum BHB levels (*n* = 10 per group). (**E**) SPT results, quantified as the percentage of sucrose solution consumed relative to total liquid intake (*n* = 10 per group). (**F**) CPP test results, expressed as mean time spent in the reward-paired chamber (t_cs_) relative to total time in both chambers (t_cs_ + t_us_), where t_cs_ and t_us_ represent time spent in conditioned and unconditioned sides (*n* = 10 per group). (**G**) CPP preference score, calculated as (t_cs_ − t_us_)/(t_cs_ + t_us_) (*n* = 10 per group). (**H**) Representative movement trajectories of mice from both groups during the CPP test. (**I**) Percentage of time spent in the center zone in the OFT (*n* = 10 per group). (**J**) Total distance traveled in the OFT (*n* = 10 per group). (**K**) Representative locomotion paths in the OFT. Data were presented as the mean ± SD from *n* = 10 mice per group. * *p* < 0.05, ** *p* < 0.01, *** *p* < 0.001 indicate significant differences between the KD and ND groups (Student’s *t*-test with FDR correction). “Mouse Icon” created by BioRender (https://www.biorender.com/icon/mouse-icon). were used in (**A**). Accessed on: 26 December 2025.

To investigate the effects of a KD on reward perception and related behavioral phenotypes in mice, we first established a KD mouse model (Figure 1A). Eight-week-old specific pathogen-free (SPF) male C57BL/6J mice were selected to minimize potential influences from growth hormones and estrogen. The mice were randomly assigned to either the KD group or the normal diet (ND) group. The KD group received a high-fat, adequate-protein, and very low-carbohydrate KD, while the ND group was fed a standard chow diet for 10 weeks. Body weight and food intake were monitored regularly throughout the intervention. During the modeling period, behavioral tests, including the sucrose preference test (SPT), conditioned place preference (CPP) test, and open field test (OFT), were conducted. Body weight monitoring revealed no significant difference between the two groups at baseline. Compared with the ND group, the KD group exhibited a slightly lower rate of weight gain, resulting in significantly lower body weight in the KD group (t(18) = 2.50, *p* < 0.05) by the end of the 10-week period (Figure 1B) [32]. Analysis of food intake revealed no significant difference in average daily consumption between the KD and ND groups (t(18) = 0.85, *p* = 0.41, Figure 1C), indicating that the KD did not markedly affect basal appetite and ruling out potential confounding effects of dietary intake on subsequent behavioral outcomes. At the end of the intervention, serum levels of the ketone body beta-hydroxybutyrate (BHB) were measured. Compared with the ND group, the KD group presented a significant increase in serum BHB (t(18) = 5.12, *p* < 0.001), confirming the successful induction of the ketogenic state (Figure 1D).

The sucrose preference test (SPT), a classic method for assessing reward perception in animals, measures the preference for sweet taste to reflect reward sensitivity [33]. Compared with ND-fed mice, KD-fed mice exhibited a significantly greater sucrose preference rate (t(18) = 3.45, *p* < 0.01, Figure 1E), suggesting that the KD increased subjective pleasure derived from natural rewards and increased reward perception. The conditioned place preference (CPP) test [34] further validated the modulatory effect of KD on reward processing. During the training phase, the reward stimuli were paired with a specific context. In the test phase, the time spent in the reward-paired chamber was recorded. ND mice spent no significant difference in time between the reward-paired and unpaired chambers. In contrast, compared with the ND mice, the KD mice spent significantly more time in the reward-paired chamber than in the unpaired side (t(18) = 4.01, *p* < 0.001, Figure 1F–H). These results demonstrate that the KD enhances associative learning for conditioned rewards, further supporting an enhanced reward phenotype. The open field test (OFT) was used to evaluate spontaneous locomotion, exploratory behavior, and anxiety-like behavior. Compared with ND mice, KD mice spent a significantly greater percentage of time in the central zone (t(18) = 2.98, *p* < 0.01, Figure 1I), indicating increased exploratory drive, potentially linked to reduced anxiety-like behavior. However, the total distance travelled did not differ significantly between the two groups (t(18) = 0.67, *p* = 0.51, Figure 1J,K), indicating that the KD did not affect basal locomotor activity and ruling out motor function as a confounding factor in reward-related behavioral outcomes. Collectively, these results demonstrated that the KD significantly enhanced reward perception in mice.

### 2.2. The Ketogenic Diet Remodeled the Hippocampal Proteomic Profile in Mice

**Figure 2:**
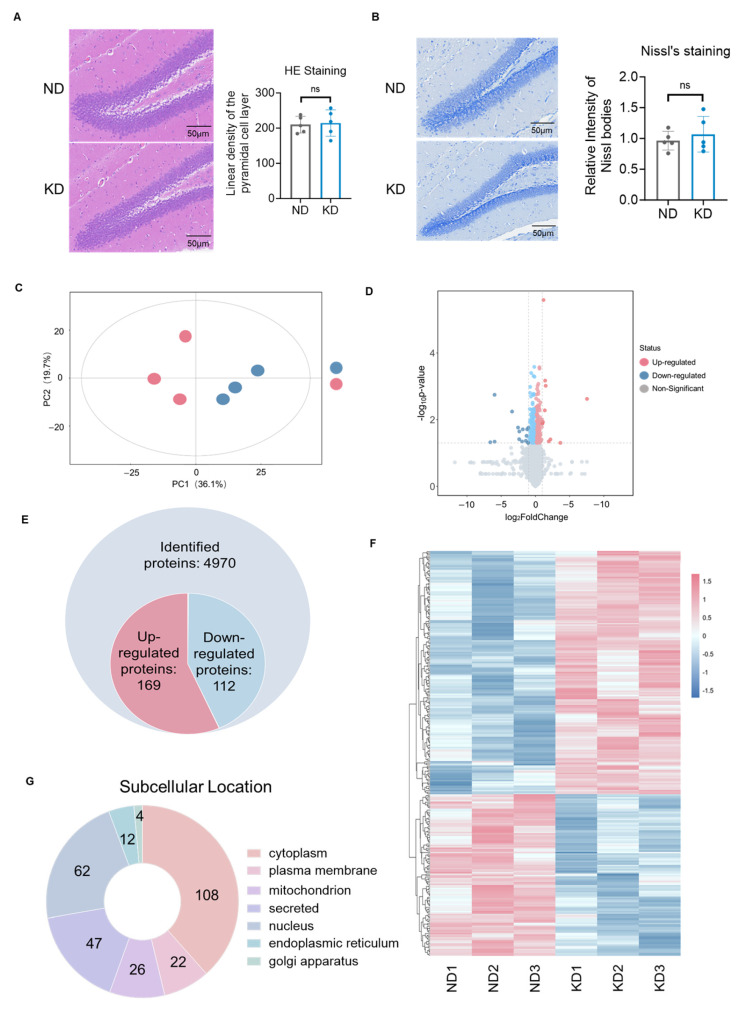
Ketogenic diet remodeled the hippocampal proteomic landscape. (**A**) Representative images of Hematoxylin and Eosin (HE) staining and quantification of the linear density of the pyramidal cell layer (number of cells per 100 μm) in the hippocampal DG region from ND and KD mice (*n* = 5 per group). (**B**) Representative images of Nissl staining and quantification of the relative intensity of Nissl bodies in the hippocampal from ND and KD mice (*n* = 5 per group). Scale bar = 50 μm. (**C**) PCA score plot of hippocampal proteomes. (**D**) Volcano plot of DEPs. (**E**) Venn diagram summarizing quantified and DEPs. (**F**) Hierarchical clustering of the 280 DEPs. (**G**) Subcellular localization analysis of DEPs. Proteins with fold change > 1.5 and *p* < 0.05 (Student’s *t*-test with FDR correction) were considered differentially expressed. Data represent the mean ± SD from *n* = 3 biological replicates per group.

To investigate the mechanism underlying KD-enhanced reward perception, we first assessed whether the KD induced any structural damage or neuronal loss in the hippocampus, as such alterations could confound the results of the behavioral and molecular analyses. Hematoxylin and eosin (HE) staining revealed no significant morphological differences in the hippocampal DG regions between the ND and KD groups, with intact cell arrangement and clear layering observed in both groups (t(8) = 0.35, *p* = 0.74, Figure 2A). Similarly, Nissl staining revealed no significant changes in the number of Nissl bodies or neuronal density in the KD group compared with the ND group (t(8) = 0.42, *p* = 0.70, Figure 2B), indicating that the KD did not cause overt neurotoxicity or neuronal loss. These results suggest that the behavioral and metabolic changes induced by the KD are not attributable to gross structural damage to hippocampal neurons.

To further elucidate the molecular mechanisms by which the KD modulated hippocampal function, we performed an untargeted proteomic analysis of hippocampal tissues from mice in each group. PCA revealed distinct clustering between the KD and ND groups with tight intragroup aggregation (Figure 2C), indicating significant differences in global protein expression profiles. The distribution of differentially expressed proteins (DEPs) was visualized by a volcano plot (|log_2_(fold change)| > 1 and *p* < 0.05 (Student’s *t*-test), Figure 2D). Proteomic analysis revealed 4970 credible proteins, among which 280 were significantly differentially expressed, comprising 169 upregulated and 112 downregulated proteins (Figure 2E). Heatmap analysis further demonstrated a distinct clustering pattern of these 280 DEPs across sample groups (Figure 2F), confirming that the KD significantly remodeled the hippocampal protein expression network. Subcellular localization analysis of the 280 differentially expressed proteins revealed a distinct distribution pattern, with predominant enrichment in the cytoplasm, followed by secretory pathway and nuclear proteins (Figure 2G). The marked cytoplasmic accumulation suggests that the KD might modulate hippocampal cellular physiology by altering key functional proteins in this central hub of metabolism and signaling. The substantial proportion of secretory proteins further suggests their potential involvement in regulating intercellular communication and synaptic homeostasis, which is consistent with our metabolomics findings demonstrating dopaminergic pathway activation. Together, the proteomic data revealed that KD substantially remodeled the hippocampal proteome, with cytoplasmic and secretory pathways emerging as functional hotspots potentially underlying dopaminergic synaptic modulation.

### 2.3. Proteomic Enrichment Analysis Identified the Dopaminergic Synapse as a Key Pathway

**Figure 3:**
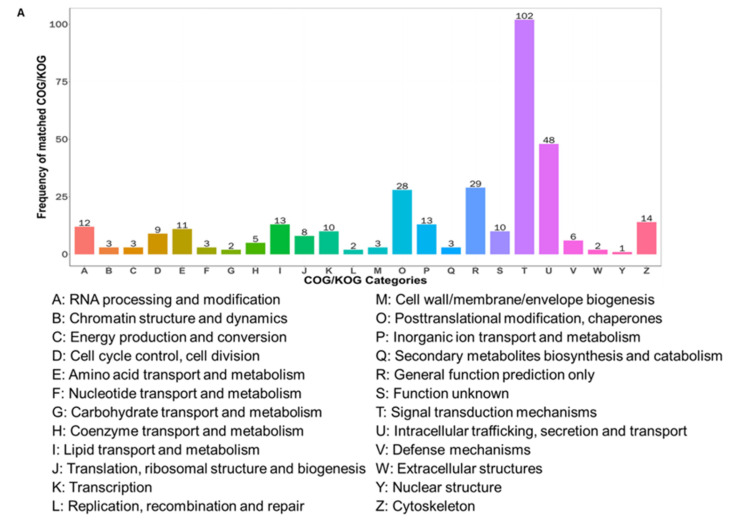

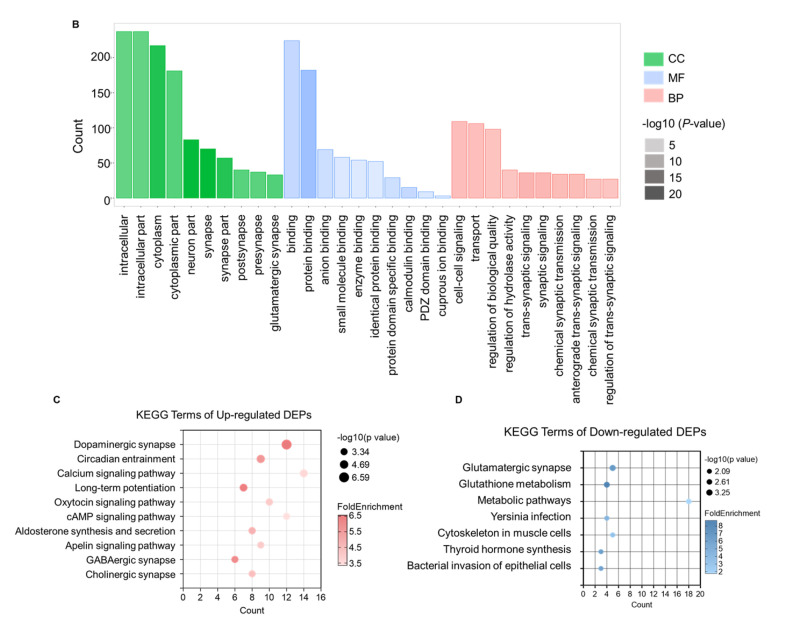
Proteomic enrichment analysis identified the dopaminergic synapse as a key regulated pathway. (**A**) COG classification of DEPs. (**B**) GO enrichment analysis of DEPs (top 10 terms per category). (**C**) KEGG pathway enrichment analysis of upregulated DEPs, highlighting the dopaminergic synapse. (**D**) KEGG pathway enrichment analysis of downregulated DEPs. Pathways with *p* < 0.05 (Fisher’s exact test with FDR correction) were considered statistically significant. All enrichment analyses were performed using the 280 DEPs identified between the KD and ND groups. Data represent the mean ± SD from *n* = 3 biological replicates per group.

To elucidate the mechanisms through which the KD modulates hippocampal protein expression and increases reward learning, we performed functional enrichment analysis of the DEPs. Clusters of Orthologous Groups (COG) classification revealed predominant involvement in “signal transduction mechanisms” and “intracellular trafficking, secretion, and vesicular transport” (Figure 3A), suggesting broad effects on neuronal signaling and neurotransmitter transport. Gene Ontology (GO) analysis further demonstrated DEP enrichment in terms of intracellular, cytoplasmic, neuronal, and synaptic components; protein and anion binding functions; and biological processes, including intercellular and trans-synaptic signaling (Figure 3B), reinforcing the role of the KD in modulating synaptic communication. KEGG pathway analysis of the upregulated DEPs revealed significant enrichment in the dopaminergic synapse pathway, along with calcium signaling, long-term potentiation, and GABAergic synapse pathways (Figure 3C). Conversely, the downregulated DEPs were enriched in the glutamatergic synapse and glutathione metabolism pathways (Figure 3D), indicating shifts in the excitatory–inhibitory balance and antioxidative responses. Together, these proteomic findings indicate that the KD remodeled hippocampal protein expression, predominantly affecting synaptic signaling and vesicular transport, with specific activation of the dopaminergic synapse pathway, providing key molecular insights into how the KD may enhance reward learning.

### 2.4. Metabolomics Revealed Activation of the Hippocampal Dopaminergic Synapse Pathway by the KD

**Figure 4:**
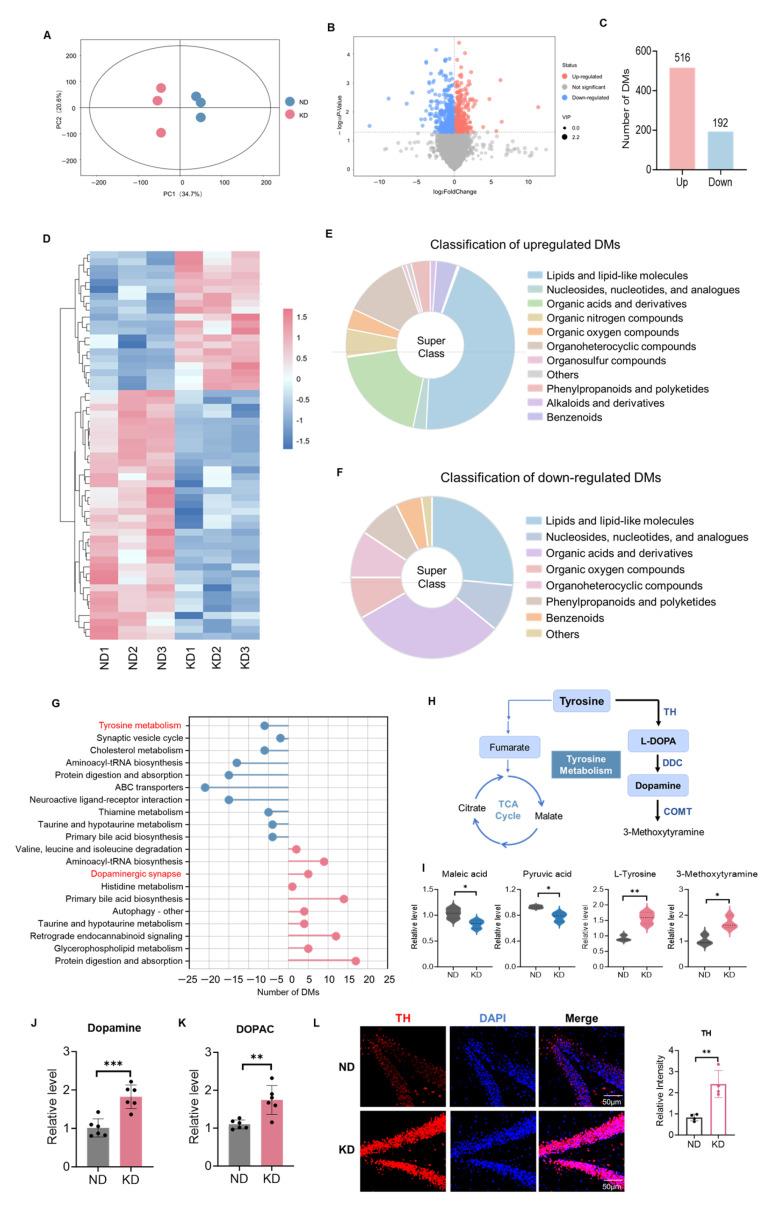
Metabolomics Revealed Activation of the Hippocampal Dopaminergic Synapse Pathway by the KD. (**A**) PCA score plot of metabolic profiles. (**B**) Volcano plot of differentially abundant metabolites. (**C**) Number of significantly upregulated and downregulated metabolites. (**D**) Heatmap of differential metabolite expression. (**E**,**F**) Chemical category classification of upregulated (**E**) and downregulated (**F**) metabolites. (**G**) Top 20 enriched KEGG pathways for differential metabolites. Red color indicated the pathways related to dopamine and tyrosine metabolism. (**H**) Schematic representation of tyrosine metabolism pathway. (**I**) Normalized abundances of selected metabolites related to tyrosine metabolism and dopaminergic synapse pathways.(* *p* < 0.05, ** *p* < 0.01) (**J**,**K**) ELISA quantification of dopamine (*** *p* < 0.001, (**J**)) and DOPAC (** *p* < 0.01, (**K**)) in hippocampal tissues. Both were significantly elevated in KD vs. ND mice (*n* = 6 per group; *t*-test). Data are the mean ± SD. (**L**) Tyrosine hydroxylase (TH) immunofluorescence in the dentate gyrus. KD mice showed significantly higher TH intensity than ND mice (*n* = 4 per group; ** *p* < 0.01, *t*-test). Scale bar: 50 μm. Metabolites with variable importance in projection (VIP) > 1.0 and *p* < 0.05 were considered statistically significant. Data are presented the as mean ± SD from *n* = 3 mice per group.

Given that our proteomic analysis revealed significant remodeling of hippocampal synaptic proteins, particularly the upregulation of the dopaminergic synapse pathway, we next sought to investigate the upstream metabolic changes that may drive these proteomic adaptations. Therefore, we performed untargeted metabolomic analysis on hippocampal tissues from ND and KD mice. We performed untargeted metabolomic analysis on samples from each group. Principal component analysis (PCA) revealed clear separation between the KD and ND groups in the score plot (Figure 4A), indicating significant differences in the overall metabolic profiles of hippocampal tissues and good intragroup reproducibility, providing a reliable basis for subsequent screening of differentially abundant metabolites. A volcano plot visually displays the distribution of these differentially abundant metabolites between the two groups (|log_2_(fold change)| > 1 and *p* < 0.05 (Student’s *t*-test), Figure 4B). Statistical analysis revealed 708 significantly altered metabolites, with 516 significantly upregulated and 192 significantly downregulated (Figure 4C). Heatmap analysis further confirmed the distinct clustering patterns of these differentially abundant metabolites, clearly distinguishing the metabolic expression profiles of the KD and ND groups (Figure 4D), suggesting that KD significantly remodeled the metabolic network in the hippocampus. Classification and annotation of the differentially abundant metabolites revealed that lipids and lipid-like molecules, along with organic acids and derivatives, were the most abundant categories among the upregulated metabolites (Figure 4E). Other categories included nucleotides and derivatives, organo-oxygen compounds, and organic nitrogen compounds. The downregulated metabolites exhibited a similar distribution pattern, with lipids and organic acids remaining the predominant altered categories (Figure 4F). These results indicate that the KD predominantly influences hippocampal lipid and organic acid metabolism, which aligns with its known metabolic properties.

To identify the core biological pathways involving the differentially expressed metabolites, we performed KEGG pathway enrichment analysis on the up- and downregulated metabolites. The top 10 enriched pathways were primarily associated with synaptic transmission, metabolic synthesis, and signal transduction. Notably, the dopaminergic synapse pathway was significantly enriched and predominantly upregulated, whereas tyrosine metabolism was significantly downregulated (Figure 4G). Tyrosine, as an important precursor for dopamine synthesis [35], is metabolized mainly in the body through two pathways: one is to generate fumarate through multiple reactions and enter the tricarboxylic acid cycle, and the other is to be catalyzed by tyrosine hydroxylase to form L-Dopa, which is then converted into dopamine by Dopa-decarboxylase (DDC) (Figure 4H) [36,37]. Further analysis of metabolites associated with the dopaminergic synapse and tyrosine metabolism pathways revealed that the levels of both tyrosine and 3-methoxytyramine (3-MT), a specific dopamine metabolite, were significantly greater in the KD group than in the control group (tyrosine: t(4) = 4.12, *p* < 0.01; 3-MT: t(4) = 2.98, *p* < 0.05, Figure 4I). In contrast, key TCA cycle intermediates such as malate and pyruvate were significantly downregulated (malate: t(4) = 2.87, *p* < 0.05; pyruvate: t(4) = 3.05, *p* < 0.05, Figure 4I). These findings suggest that under KD conditions, tyrosine metabolism preferentially shifts toward dopamine synthesis rather than energy production via the TCA cycle. To directly assess dopamine content and turnover, we performed ELISA assays on hippocampal tissue. Both dopamine and its primary metabolite DOPAC (3,4-dihydroxyphenylacetic acid) were significantly elevated in the KD group compared with the ND group (dopamine: t(10) = 4.56, *p* < 0.001; DOPAC: t(10) = 3.87, *p* < 0.01, Figure 4J,K). Furthermore, tyrosine hydroxylase (TH) immunofluorescence in the dentate gyrus (DG) region revealed that KD mice exhibited significantly stronger TH fluorescence intensity than ND mice (t(6) = 5.12, *p* < 0.001, Figure 4L), indicating increased dopaminergic fiber density or terminal arborization. Collectively, these results demonstrate that the KD remodeled the hippocampal metabolic network and activated the dopaminergic synaptic pathway by redirecting tyrosine metabolic flux, with complementary ELISA and immunohistochemical evidence directly confirming enhanced dopaminergic signaling.

### 2.5. Integrated Multiomics Analysis Identified CAMK2A as a Central Node in the Dopaminergic Pathway

**Figure 5:**
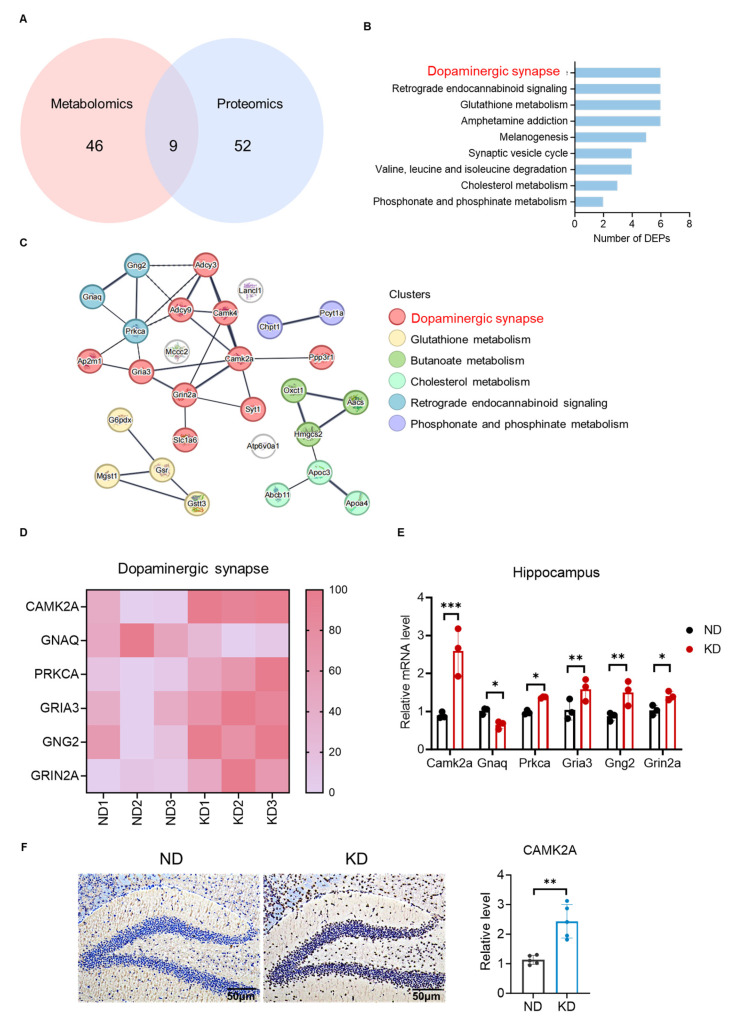
Integrated multi-omics analysis identified CAMK2A as a central node in the dopaminergic pathway. (**A**) Venn diagram of overlapping KEGG pathways from metabolomic and proteomic analyses. (**B**) Significance ranking of the overlapping pathways. (**C**) PPI network of DEPs within the overlapping pathways. (**D**) Heatmap of key protein expression in the dopaminergic pathway. (**E**) RT-qPCR validation of key gene expression in the dopaminergic pathway (*n* = 3 per group). (**F**) Representative immunohistochemical images of CAMK2A expression in the hippocampal DG region from ND and KD mice and quantification of the relative level of CAMK2A immunostaining (*n* = 3 per group). Scale bar = 50 μm. Pathway enrichment significance was determined by Fisher’s exact test with FDR correction (*p* < 0.05). Protein expression changes are shown as log2 fold change (KD/ND). RT-qPCR data are presented as the mean ± SD from *n* = 3 biological replicates per group (* *p* < 0.05, ** *p* < 0.01, *** *p* < 0.001, Student’s *t*-test).

To comprehensively assess the systemic impact of the KD on the hippocampus at both the metabolic and proteomic levels, we integrated the results of the KEGG pathway enrichment analyses. Metabolomics revealed 46 enriched pathways, whereas proteomics revealed 52, with intersection analysis identifying 9 overlapping pathways (Figure 5A). These shared pathways, which were significantly altered in both datasets, represented key coordinated targets of KD intervention in hippocampal function. When the genes were ranked by statistical significance, the enrichment of the dopaminergic synapse pathway was significantly greater among the 9 overlapping pathways (Figure 5B). Other shared pathways included retrograde endocannabinoid signaling, the synaptic vesicle cycle, and cholesterol metabolism. This integrated analysis strongly implicated the dopaminergic synapse pathway as a central signaling mechanism through which KD modulated hippocampal function.

To further identify key regulatory proteins, we constructed a protein–protein interaction (PPI) network using the STRING database. Network analysis revealed CAMK2A as a central hub within the dopaminergic synapse pathway, demonstrating extensive interactions with other pathway components (Figure 5C). These findings suggest that CAMK2A is a potential pivotal regulator of the dopaminergic signaling network. We further examined the expression levels of all DEPs associated with the dopaminergic synapse pathway. Heatmap analysis was used to visualize the expression patterns of six key DEPs in the KD group versus the ND group. GNAQ was downregulated, while five proteins—CAMK2A, PRKCA, GRIA3, GNG2 and GRIN2A—were significantly upregulated (Figure 5D). The differential expression of each protein was statistically significant (CAMK2A: t(4) = 5.12, *p* < 0.001; GNAQ: t(4) = 2.67, *p* < 0.05; PRKCA: t(4) = 3.21, *p* < 0.05; GRIA3: t(4) = 3.98, *p* < 0.01; GNG2: t(4) = 3.45, *p* < 0.01; GRIN2A: t(4) = 2.89, *p* < 0.05). This coordinated expression profile, particularly the upregulation of CAMK2A, strongly indicates specific activation of this pathway by KD. To validate these proteomic findings at the transcriptional level, we quantified the hippocampal mRNA expression of these six genes using RT–qPCR. The results were fully consistent with the proteomic data (Figure 5E). Again, CAMK2A showed the most pronounced upregulation (t(4) = 5.89, *p* < 0.001), with similar significant changes observed for the other five genes (all t(4) > 2.78, *p* < 0.05). Notably, the expression of CAMK2A was most strongly upregulated at the mRNA level, further supporting its role as the most responsive molecular component of the KD-modulated dopaminergic pathway. To further corroborate these findings at the protein level and with spatial resolution, we performed immunohistochemical staining for CAMK2A in hippocampal tissue sections. Representative images and quantitative analysis of the mean optical density revealed significantly greater CAMK2A immunoreactivity in the KD group than in the ND group, particularly in the DG region (Figure 5F). Quantification confirmed a significant increase in CAMK2A immunoreactivity in the KD group compared with the ND group (t(6) = 3.89, *p* < 0.01). These results provide orthogonal validation at the tissue level, confirming that CAMK2A upregulation occurs consistently across proteomic, transcriptomic, and histological analyses. In summary, integrated multiomics and histological analyses revealed the dopaminergic synapse pathway as a central target of KD modulation, with CAMK2A emerging as its key regulatory node, which was consistently upregulated at the protein, mRNA, and tissue levels under KD conditions.

### 2.6. BHB Derived Dopaminergic Synaptic Signaling Through CAMK2A Upregulation

**Figure 6:**
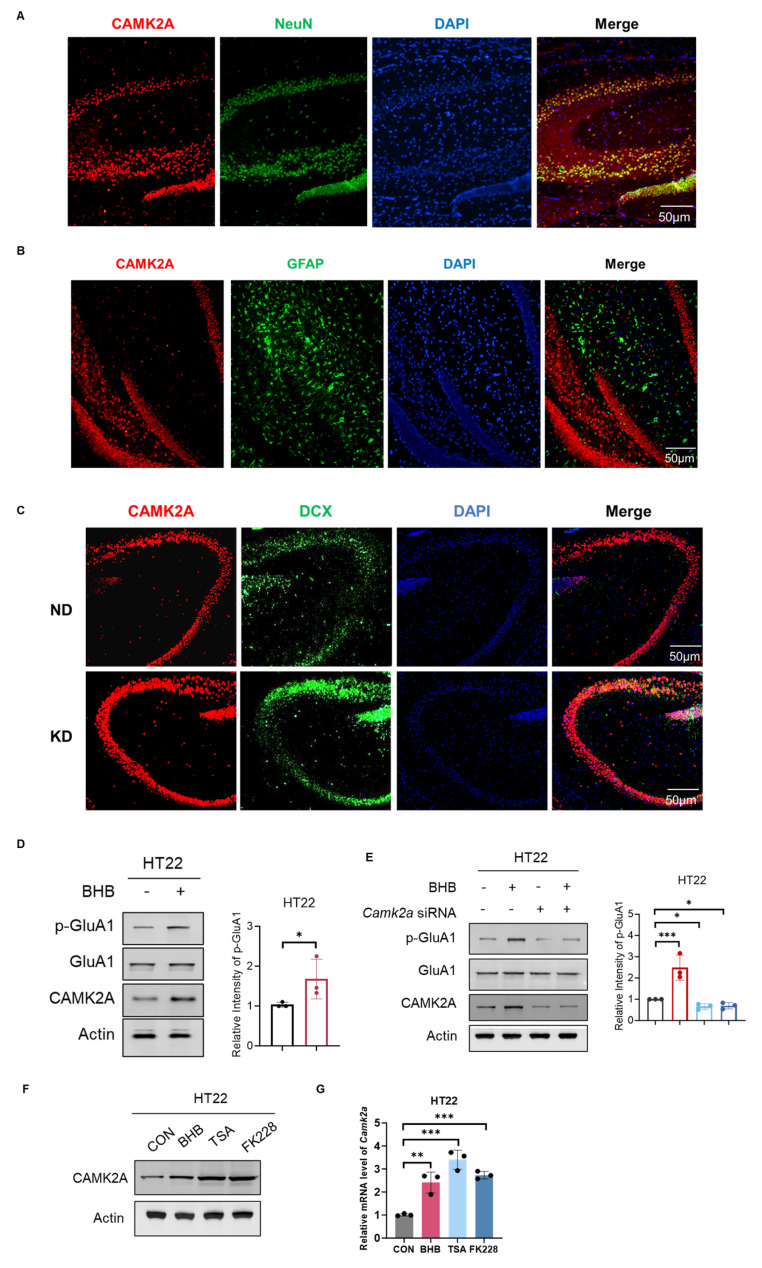
BHB derived dopaminergic synaptic signaling through CAMK2A upregulation. (**A**) Immunofluorescence of CAMK2A (red) and the neuronal marker NeuN (green) in hippocampus (*n* = 3 independent experiments). (**B**) Immunofluorescence of CAMK2A (red) and the astrocytic marker GFAP (green) (*n* = 3 independent experiments). (**C**) Immunofluorescence of CAMK2A (red) and DCX (green) in KD and ND groups (*n* = 3 independent experiments). (**D**) Western blot analysis (left) and quantitative analysis (right) of CAMK2A, GluA1, and p-GluA1 levels in BHB-treated HT22 cells (*n* = 3 independent experiments). (**E**) Western blot analysis (left) and quantitative analysis (right) of p-GluA1 after *Camk2a* siRNA knockdown in BHB-treated cells (*n* = 3 independent experiments). Scale bars: 50 μm. (**F**) Western blot analysis of CAMK2A protein levels. (**G**) RT-qPCR analysis of *CAMK2A* mRNA levels, normalized to β-actin (*n* = 3 independent experiments). Data represent the mean ± SD of three independent experiments (* *p* < 0.05, ** *p* < 0.01, *** *p* < 0.001, Student’s *t*-test).

To determine the cellular localization of CAMK2A in hippocampal tissue and the effect of KD on its expression, we first performed immunofluorescence staining. The results revealed significant colocalization of the CAMK2A protein with the neuronal marker NeuN (Figure 6A), confirming its predominant expression in neurons. In contrast, no apparent colocalization signal was observed between CAMK2A and the astrocyte marker GFAP (Figure 6B), ruling out its primary expression in glial cells. Compared with ND mice, KD mice exhibited significantly greater CAMK2A fluorescence intensity in hippocampal neurons (t(4) = 3.45, *p* < 0.01, Figure 6C), providing further in situ protein-level confirmation that the KD upregulated CAMK2A expression in neurons. Concurrently, the fluorescence intensity of the neural precursor cell marker DCX increased in the KD group, suggesting that the KD may promote hippocampal neurogenesis or plasticity.

BHB, the primary ketone body produced during ketosis, serves as a key effector molecule that mediates the neurological effects of the KD [13]. To verify whether BHB directly regulates CAMK2A, we treated the mouse hippocampal neuronal cell line HT22 with BHB. Western blot analysis revealed that BHB treatment significantly upregulated the CAMK2A protein levels in HT22 cells (t(4) = 4.12, *p* < 0.01, Figure 6D). We further investigated the phosphorylation state of GluA1, a key AMPA receptor subunit critically involved in dopaminergic synaptic signaling and long-term potentiation (LTP) [38]. While total GluA1 protein levels remained unchanged, BHB treatment markedly increased its phosphorylation (p-GluA1) (t(4) = 3.21, *p* < 0.05), indicating that BHB not only upregulated CAMK2A but also activated downstream synaptic plasticity-related signaling. To determine whether CAMK2A is required for BHB-mediated signaling, we knocked-down *Camk2a* expression in HT22 cells. Following *Camk2a* depletion, BHB treatment no longer increased the p-GluA1 levels (siControl + BHB vs. siCamk2a + BHB: t(4) = 0.45, *p* = 0.67, Figure 6E), demonstrating that the increase in p-GluA1 induced by BHB is strictly dependent on CAMK2A. To elucidate the mechanism linking BHB to CAMK2A upregulation, we investigated its potential role as an endogenous HDAC inhibitor. Building on prior evidence that BHB inhibits Class I HDACs [13,25], we conducted in vitro experiments and demonstrated that treatment with BHB, the pan-HDAC inhibitor Trichostatin A (TSA), or the HDAC1/2-specific inhibitor Romidepsin (FK228) significantly increased CAMK2A expression at both the mRNA and protein levels (BHB: t(4) = 3.98, *p* < 0.01; TSA: t(4) = 4.23, *p* < 0.001; FK228: t(4) = 4.05, *p* < 0.001, Figure 6F,G). Collectively, these findings indicate that BHB, the key KD metabolite, directly acts on neurons to upregulate CAMK2A expression, thus driving downstream signaling events, including increased GluA1 phosphorylation, through a CAMK2A-dependent cascade.

### 2.7. Hippocampal CAMK2A Was Required for the KD to Enhance Reward Perception

**Figure 7:**
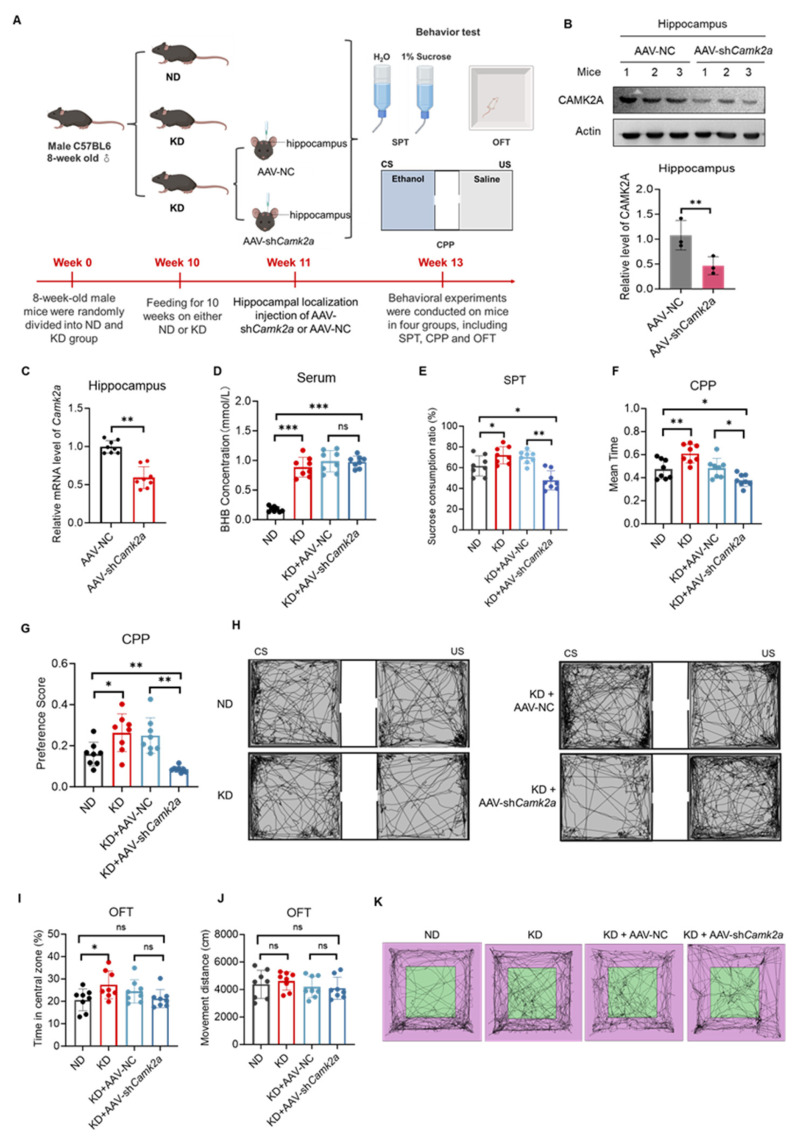
Hippocampal *Camk2a* knockdown attenuated KD-enhanced reward perception. (**A**) Experimental design schematic showing the four groups (ND, KD, KD + AAV-NC, and KD + AAV-sh*Camk2a*) and stereotaxic targeting of the dorsal hippocampus. (**B**) Western blot and quantitative analysis of CAMK2A in the hippocampus (*n* = 3 per group). (**C**) RT-qPCR of *Camk2a* mRNA levels in hippocampal tissues (*n* = 8 per group) (**D**) Serum BHB levels across experimental groups (*n* = 8 per group). (**E**) SPT results (*n* = 8 per group). (**F**) CPP results (time in reward-paired chamber) (*n* = 8 per group). (**G**) CPP preference score (*n* = 8 per group). (**H**) Representative movement trajectories from the CPP. (**I**) OFT results showing center zone occupancy time (*n* = 8 per group). (**J**) Total distance traveled in the OFT (*n* = 8 per group). (**K**) Representative locomotion paths from the OFT. All behavioral data are presented as the mean ± SD. * *p* < 0.05, ** *p* < 0.01, *** *p* < 0.001 indicate significant differences (Student’s *t*-test). “Mouse Icon” created by BioRender (https://www.biorender.com/icon/mouse-icon) were used in (**A**). Accessed on: 28 December 2025.

To determine the role of CAMK2A in the reward-enhancing effects of the KD in vivo, we delivered an adeno-associated virus carrying *Camk2a*-specific short hairpin RNA (AAV-sh*Camk2a*) into the hippocampus of KD-fed mice via stereotaxic injection to achieve hippocampus-specific knockdown of *Camk2a* (Figure 7A). Because the pro reward phenotype was induced specifically by KD, the necessity of CAMK2A was tested only in the KD context; ND mice served as baseline controls without intervention. Western blot analysis of hippocampal tissue confirmed a significant reduction in the CAMK2A protein levels in mice injected with AAV-sh*Camk2a* compared with those receiving AAV-NC (t(4) = 5.21, *p* < 0.01), confirming the efficacy of the knockdown (Figure 7B). RT-qPCR confirmed a significant reduction in *Camk2a* mRNA levels in the hippocampus of the AAV-sh*Camk2a* group (t(4) = 4.85, *p* < 0.01, Figure 7C). We further measured the serum BHB levels and observed the expected significant increase in KD-fed mice. Notably, hippocampal *Camk2a* knockdown did not alter the serum BHB concentrations (one-way ANOVA: F(2,21) = 15.32, *p* < 0.001 for diet effect; post hoc: KD + AAV-NC vs. KD + AAV-shCamk2a, *p* = 0.68, Figure 7D), indicating that CAMK2A acts downstream of BHB and that its behavioral effects are not mediated by changes in the systemic metabolic state.

To evaluate the effect of CAMK2A on reward learning, we first performed the SPT. Hippocampal *Camk2a* knockdown (KD + AAV-sh*Camk2a*) significantly suppressed the KD-induced increase in sucrose preference (one-way ANOVA: F(2,21) = 8.45, *p* < 0.01; post hoc: KD + AAV-NC vs. KD + AAV-sh*Camk2a*, *p* < 0.01, *p* < 0.01, Figure 7E). Similarly, the results of the CPP test demonstrated that *Camk2a* knockdown completely blocked the formation of reward-associated memory (for mean time: one-way ANOVA: F(2,21) = 10.23, *p* < 0.01; post hoc: KD + AAV-NC vs. KD + AAV-sh*Camk2a*, *p* < 0.05; for preference score: F(2,21) = 9.56, *p* < 0.05; post hoc: *p* < 0.01, Figure 7F–H). To exclude potential confounding effects of motor function, we conducted the OFT. *Camk2a* knockdown did not affect either the time spent in the center zone (one-way ANOVA: F(2,21) = 1.23, *p* = 0.31, Figure 7I) or the total distance travelled (one-way ANOVA: F(2,21) = 0.54, *p* = 0.59, Figure 7J,K), indicating that neither the KD nor CAMK2A manipulation altered anxiety-like behavior or basal locomotor activity. In summary, hippocampal *Camk2a* knockdown effectively reversed the enhancing effects of the KD on both natural and conditioned reward perception, independent of changes in fundamental motor function. These results establish CAMK2A as an indispensable molecular mediator through which the KD enhances the reward learning capacity.

## 3. Discussion

The KD has demonstrated remarkable clinical efficacy since its introduction in 1921 for epilepsy management [39]. Recent studies have further revealed its therapeutic potential for various neurological conditions [40], including cognitive enhancement and Parkinson’s disease, because of its unique metabolic profile [10,41]. In this study, we demonstrated through behavioral experiments that the KD enhanced the reward learning capacity. This study systematically elucidated a novel pathway through which KD enhanced reward learning: KD upregulated CAMK2A expression in hippocampal neurons, thus activating local dopaminergic synaptic signaling and improving reward-related behaviors. Our findings not only provide a novel mechanistic explanation for the neuromodulatory effects of the KD but also establish crucial connections between energy metabolism, synaptic plasticity, and complex behavioral outcomes.

First, our integrated unbiased proteomic and metabolomic analyses, conducted as an exploratory screening phase, provide key systems-level evidence supporting this pathway. Both COG and GO analyses of the DEPs consistently revealed “signal transduction” and “synaptic function” as central processes affected by the KD. Crucially, KEGG pathway enrichment and cross-omics integration unanimously revealed the dopaminergic synapse as the most significantly altered pathway. These findings carry dual significance: first, the findings directly link the cognitive benefits of the KD to a core reward-related pathway; second, the findings challenge the conventional reward research paradigm focused predominantly on the VTA-NAc circuit, highlighting the independent and essential role of hippocampal dopamine signaling in integrating metabolic states with reward learning. This initial screening, while based on a limited sample size, pinpointed CAMK2A as the central hub within this pathway, providing a precise target for subsequent hypothesis-driven and mechanistic investigation.

While CAMK2A is well-known as a master regulator of neuroplasticity in learning and memory [27,42], our study revealed that it is a potential critical upstream mediator through which the KD modulates reward behavior. At the cellular level, BHB directly upregulated CAMK2A expression in neurons and increased the phosphorylation of its downstream substrate GluA1—an effect that was abolished upon *Camk2a* knockdown. To explore the mechanism by which BHB upregulates CAMK2A, we treated HT22 cells with the HDAC inhibitors TSA (pan-HDAC inhibitor) and FK228 (HDAC1/2-specific inhibitor). Both inhibitors significantly increased CAMK2A expression at both the protein and mRNA levels (Figure 6F,G), phenocopying the effect of BHB. Collectively, these results support a plausible model in which BHB enhances CAMK2A transcription, at least in part, through HDAC inhibition and subsequent epigenetic regulation. While these findings provide supporting evidence that BHB may function not only as an energy substrate but also as a signaling molecule capable of engaging in a CAMK2A-dependent cascade, we acknowledge that the precise upstream molecular events linking BHB to HDAC inhibition and CAMK2A upregulation remain to be fully elucidated. Nonetheless, these findings offer new experimental insight into the nonmetabolic functions of ketone bodies in the central nervous system and provide a foundation for future mechanistic studies.

A key contribution of this study lies in our rigorous in vivo experimental validation of the behavioral necessity of hippocampal CAMK2A. Through hippocampus-specific knockdown of *Camk2a* in KD-fed mice, we successfully reversed the diet-induced increase in reward perception in both the SPT and CPP tests. These results demonstrate that despite the systemic metabolic alterations induced by the KD, the pro-reward effects observed in our study require hippocampal CAMK2A expression. Furthermore, the OFT results ruled out changes in locomotor activity or anxiety levels as alternative explanations, thus supporting a specific role for CAMK2A in reward processing. However, we acknowledge that the increased time spent in the center of the open field may also reflect reduced anxiety-like behavior or altered exploratory drive, factors that could confound the observed behavioral phenotypes. Although the open field test data help rule out general locomotor impairments, they do not fully dissociate reward sensitivity, motivation, anxiety-like behavior, and exploration. We therefore interpret our findings with caution. The collective evidence—including the selective increase in sucrose preference in the absence of changes in total fluid intake, the robust conditioned place preference effect accompanied by clear learning curves, and the absence of general locomotor alterations—suggests a predominant role for enhanced reward learning and reward sensitivity, while contributions from altered anxiety or exploration cannot be entirely excluded. Future studies incorporating more refined behavioral paradigms, such as progressive ratio testing to assess motivation or the elevated plus maze to evaluate anxiety, would help further disentangle these behavioral components. Importantly, while these knockdown experiments establish the necessity of hippocampal CAMK2A for the behavioral effects of the KD, they do not by themselves resolve the broader circuit-level contributions of hippocampal signaling to reward-related behavior, nor do they exclude the potential involvement of other brain regions. As such, our findings provide evidence for the central role of the hippocampal CAMK2A axis in reward learning while also highlighting the need for future studies employing complementary approaches such as circuit tracing or region-specific rescue to further delineate the neural circuitry underlying this effect.

The functional implications of modulating hippocampal dopaminergic signaling extend beyond comparisons of absolute neurotransmitter levels. The hippocampus is integral to associative learning, where it encodes the contextual and predictive relationships of rewards rather than generating primary hedonic drive [43,44]. Within this cognitive framework, the CAMK2A-dependent potentiation of local dopaminergic synapses, as identified here, can act as a high-gain gate for synaptic plasticity, selectively enhancing the salience and persistence of reward-related memories. This mechanism highlights that the efficacy of neuromodulation is derived from precise circuit function and temporal dynamics, not merely neurotransmitter abundance. Consequently, targeting the hippocampal CAMK2A–dopamine axis presents a distinct therapeutic strategy aimed at the cognitive and motivational deficits prevalent in disorders such as depression and addiction, potentially offering a means to recalibrate maladaptive reward learning without directly interfering with the mesolimbic circuitry central to reward consumption and addiction liability.

Nevertheless, several limitations should be acknowledged. First, the exclusive use of male mice, while controlling for confounding hormonal variability, precludes the assessment of potential sex-specific effects of ketogenic metabolism and its impact on dopaminergic signaling and reward learning. This represents an important constraint on the generalizability of our findings and warrants explicit investigation in future studies. Second, the proteomic and metabolomic screenings were conducted with *n* = 3 per group, a sample size common in exploratory omics studies for hypothesis generation. Although these findings were subsequently validated through targeted molecular and behavioral experiments, we note that no formal power analysis was performed at this initial stage. Therefore, the omics-derived conclusions should be interpreted as identifying robust candidate pathways rather than providing definitive quantitative evidence. Third, while we confirmed the BHB–CAMK2A–pGluA1 signaling axis and demonstrated BHB-induced the upregulation of CAMK2A transcription, the precise upstream molecular events mediating this transcriptional regulation remain unclear. We acknowledge that BHB may also influence CAMK2A through additional pathways, the delineation of which constitutes an important direction for future research. Fourth, the quantitative functional contribution of hippocampal dopamine signaling within the broader reward network requires further delineation using circuit-specific interrogation techniques. Finally, given that reward learning involves coordinated interactions across multiple brain regions, understanding how hippocampal CAMK2A signaling communicates with classic reward centers such as the VTA and NAc represents the next critical step for elucidating network-level mechanisms.

In summary, this study systematically revealed a complete signaling pathway through which the KD enhanced reward learning: its key metabolite BHB upregulated CAMK2A expression in hippocampal neurons, thus activating local dopaminergic synaptic signaling. Our work provides a mechanistic explanation for how the KD improves hippocampus-dependent reward learning, forges a crucial link between the energy metabolite BHB and higher-order brain function, and offers both a theoretical foundation and potential strategies for targeting CAMK2A to treat reward-related psychiatric disorders.

## 4. Materials and Methods

### 4.1. Cell Culture

The HT22 mouse hippocampal neuronal cell line was sourced from the Cell Bank of the Chinese Academy of Sciences Committee on Type Culture Collection. Culture conditions consisted of normal DMEM (Gibco, Thermo Fisher Scientific, Waltham, MA, USA) with 10% fetal bovine serum (Gibco, Thermo Fisher Scientific, Waltham, MA, USA), 100 U/mL penicillin, and 100 μg/mL streptomycin (Invitrogen, Thermo Fisher Scientific, Waltham, MA, USA) in a 37 °C humidified incubator with 5% CO_2_. For drug treatments, compounds were applied to the culture medium at the concentrations listed in the figure legends. Specifically, cells were treated with 5 mM beta-hydroxybutyrate (BHB) for a period of 12 h.

### 4.2. Animals

Eight-week-old male C57BL/6J mice weighing 20–22 g were sourced from GemPharmatech Co., Ltd. (Nanjing, China). Animals were housed under a 12-h light/dark cycle with unlimited access to food and water. Starting at 8 weeks of age, wild-type mice were maintained on either a normal diet (Bio-Serv, Frenchtown, NJ, USA, F0761) or a ketogenic diet (Bio-Serv, Frenchtown, NJ, USA F3666) for 10 weeks. All procedures were approved by the Animal Care and Use Committee of the Capital Institute of Pediatrics (Ethic Vote DWLL2025024) and conformed to the National Institutes of Health Guide for the Care and Use of Laboratory Animals. At the conclusion of the treatment period, mice were anesthetized with 2% isoflurane delivered by spontaneous inhalation and euthanized by cervical dislocation. Blood and tissue samples were collected at predefined time points. For behavioral tests (sucrose preference test, conditioned place preference, and open field test), each experimental group consisted of 8–10 mice. For proteomic and metabolomic analyses, *n* = 3 per group. For immunohistochemistry and Western blot validation, *n* = 3 per group. Detailed sample sizes are also provided in the respective figure legends.

Adult male C57BL/6 mice (8–10 weeks old) were anesthetized with ketamine/xylazine (100/10 mg/kg, i.p.) and positioned in a stereotaxic frame. AAV particles expressing *Camk2a*-specific short hairpin RNA (AAV2/9-hSyn1-sh*Camk2a*-EGFP) or control scrambled sequence (AAV2/9-hSyn1-scramble-EGFP) were bilaterally microinfused into the dorsal hippocampus using the following coordinates relative to Bregma: AP −2.0 mm, ML ±1.5 mm, DV −2.0 mm. Viral suspensions (1.0 μL per hemisphere) were delivered through 33-gauge Hamilton needles at an infusion rate of 0.2 μL/min. Following each injection, needles remained in place for 5 min to ensure complete diffusion and prevent reflux. All surgical procedures were performed under aseptic conditions. Mice were allowed 3 weeks for viral expression and recovery before subsequent behavioral and molecular analyses.

### 4.3. Sucrose Preference Test (SPT)

Prior to formal testing, mice will undergo a 24-h acclimation period to sucrose. Each animal will be individually housed and provided with two bottles—one containing tap water and the other 1% sucrose solution. To prevent the development of place preference, the positions of the two bottles were switched every 8 h. During the test phase, mice were again presented with both water and sucrose bottles, though without positional alternation. After 12 h, consumption volumes of both liquids were measured. Sucrose preference was calculated as follows: (sucrose intake × 100)/(sucrose intake + water intake).

### 4.4. Ethanol-Induced Conditioned Place Preference (CPP)

The ethanol-induced CPP paradigm was systematically assessed using a three-chamber apparatus (Xinruan, Shanghai, China) featuring distinct visual environments: one white chamber with circular floor patterns and one black chamber with rectangular patterns, connected by a neutral central compartment. The experimental protocol progressed through three distinct phases conducted over 10 days.

During the initial pre-test (day 1), mice were granted 20-minute free access to all chambers while SuperMaze software V2.14 (Xinruan, Shanghai, China) quantified the baseline chamber occupancy. Based on these measurements, chamber assignments were made using a counterbalanced design where the initially non-preferred chamber was designated for ethanol pairing. The conditioning phase (days 2–9) employed a standardized protocol consisting of four ethanol–saline pairing cycles. This phase followed an alternating schedule where mice received intraperitoneal administration of either 20% ethanol solution (0.25 mL in saline) or equivalent-volume saline on consecutive days, with each injection followed by 5-minute confinement in the corresponding paired chamber. The final post-test (day 10) evaluated preference formation by allowing 20-minute free chamber access without any treatment. Chamber occupancy durations were automatically recorded to quantify potential shifts in spatial preference resulting from the ethanol-chamber associations established during conditioning.

### 4.5. Open Field Test (OFT)

Mice were individually introduced into a square open-field arena (50 × 50 cm) and allowed to explore freely for a 5-minute session. The arena was digitally divided into a central zone (25 × 25 cm) and peripheral zones for automated analysis. All sessions were recorded using the SuperMaze video tracking system (Xinruan, Shanghai, China), which automatically quantified the total distance traveled and time spent in the central zone. Movement trajectories were subsequently analyzed to assess exploratory behavior and anxiety-like responses.

### 4.6. RNA Isolation and Real-Time qPCR Analyses

Total RNA was extracted from cells using the FastPure Cell/Tissue Total RNA Isolation Kit V2 (Vazyme, Nanjing, China), following the manufacturer’s recommended protocol. RNA concentration and purity were assessed using a NanoDrop Spectrophotometer (Thermo Fisher Scientific, Waltham, MA, USA). First-strand cDNA was synthesized from equal amounts of total RNA with the HiScript III 1st Strand cDNA Synthesis Kit (Vazyme, Nanjing, China) according to the supplier’s instructions. Quantitative real-time PCR was subsequently carried out using SYBR qPCR Master Mix (Vazyme, Nanjing, China) on a Real-Time Thermocycler (BioRad, Hercules, CA, USA). Gene expression levels in cells or tissues were normalized to the endogenous control *β-actin*. Primer sequences used in this study are provided in the Table S1.

### 4.7. SDS-PAGE and Western Blot

SDS-PAGE and immunoblotting were carried out according to standard laboratory procedures. Cells were lysed in a loading buffer composed of 50 mM Tris-HCl (pH 6.8), 10% glycerol, 2% SDS, 4% β-mercaptoethanol, and 0.0012% bromophenol blue. Protein samples were resolved by SDS-PAGE and subsequently transferred onto nitrocellulose membranes (GE Healthcare, Chicago, IL, USA). Membranes were blocked for 1 h at room temperature with 5% non-fat milk in TBST (Tris-buffered saline containing 0.1% Tween 20), followed by overnight incubation at 4 °C with primary antibodies diluted in antibody dilution buffer (QuickBlock™, Beyotime, Shanghai, China). After washing, membranes were incubated with HRP-conjugated secondary antibodies diluted in TBST supplemented with 5% non-fat milk. Protein signals were visualized using ECL Plus reagent (Thermo Fisher Scientific) and captured with a Typhoon scanner (GE Healthcare).

### 4.8. Metabolomics Analysis

Samples were thawed at room temperature. A 500-μL aliquot of each sample was mixed with L-2-chlorophenylalanine (0.06 mg/mL in methanol) as an internal standard. A pre-cooled mixture of methanol and acetonitrile (2:1, *v*/*v*) was added, and samples were vortexed, sonicated in an ice-water bath, and kept at −20 °C for 30 min. After centrifugation (13,000× *g* rpm, 4 °C, 10 min), 400 μL of the supernatant was dried under vacuum centrifugation. The residue was reconstituted in 100 μL of methanol–water (1:4, *v*/*v*), briefly vortexed and sonicated, then stored at −20 °C for 2 h. Following a second centrifugation, the supernatant was filtered (0.22 μm) and transferred to LC vials. Quality control samples were prepared by pooling equal aliquots from all experimental samples.

Metabolomic profiling was performed using ultra-high-performance liquid chromatography coupled with quadrupole-Orbitrap mass spectrometry (UPLC-Q-Exactive, Thermo Fisher Scientific, Waltham, MA, USA). Separation was achieved on an ACQUITY UPLC HSS T3 column (Waters Corporation, Milford, MA, USA, 1.8 μm, 2.1 × 100 mm) maintained at 45 °C. The mobile phases were water (A) and acetonitrile (B), each containing 0.1% formic acid. Gradient elution was carried out at 0.35 mL/min as follows: 5% B from 0–2 min, ramped to 30% B at 4 min, to 50% B at 8 min, to 80% B at 10 min, to 100% B at 14 min, held until 15 min, then returned to 5% B at 15.1 min and re-equilibrated until 16 min. Mass spectrometry was conducted in both positive and negative heated electrospray ionization (HESI) modes. Full MS scans were acquired over *m/z* 100–1000 at a resolution of 70,000, with data-dependent MS/MS scans performed at a resolution of 17,500 using stepped collision energies of 10, 20, and 40 eV.

LC-MS raw data were processed using Progenesis QI V2.3 (Nonlinear Dynamics, Newcastle upon Tyne, UK). Compound identification was performed by matching accurate *m/z*, MS/MS fragments, and isotopic patterns against public databases (HMDB, Lipidmaps, Metlin, La Jolla, CA, USA) and an in-house library. Peaks with >50% missing values per group were removed, and remaining missing values were imputed with half of the minimum intensity. A final dataset was generated by merging positive and negative ion mode data. PCA was used to assess the sample distribution. OPLS-DA was applied to identify differential metabolites between groups. Metabolites with variable importance in projection (VIP) > 1.0 and *p* < 0.05 (Student’s *t*-test) were considered significantly altered and subjected to KEGG pathway enrichment analysis.

### 4.9. Proteomics Analysis

Proteins were extracted from samples stored at −80 °C using lysis buffer (8 M urea, 1% protease inhibitor) followed by sonication on ice and centrifugation (12,000× *g*, 10 min, 4 °C). Supernatant protein concentrations were determined by the BCA assay. Aliquots (100 μg protein) were reduced (10 mM DTT, 56 °C, 1 h), alkylated (20 mM IAA, dark, 45 min) and digested with trypsin (1:50, *w*/*w*) overnight at 37 °C. Resulting peptides were desalted using C18 cartridges, vacuum-dried, and stored at −80 °C.

LC-MS/MS analysis was conducted on a nanoElute UHPLC system coupled to a timsTOF Pro mass spectrometer (Bruker, Billerica, MA, USA). Peptides were separated on a C18 column (75 μm × 25 cm, 1.6 μm, Agilent Technologies, Santa Clara, CA, USA) using a 90 min gradient (2–95% acetonitrile with 0.1% formic acid) at 300 nL min^−1^. Data were acquired in PASEF mode over *m*/*z* 100–1700.

Raw data were processed in MaxQuant (v2.0.3.0) using the Andromeda engine against the UniProt database. Search parameters: precursor tolerance, 10 ppm; fragment tolerance, 0.5 Da; trypsin specificity (≤2 missed cleavages). FDR was set to <1% at both the peptide and protein levels. Label-free quantification was performed using the MaxLFQ algorithm.

Differentially abundant proteins were identified by *t* test or ANOVA (*p* < 0.05, fold change > 1.5 or <0.67). PCA and hierarchical clustering were applied for sample grouping. Functional enrichment analysis (GO, KEGG) was performed using Fisher’s exact test with Benjamini–Hochberg correction (*p* < 0.05).

### 4.10. β-Hydroxybutyrate Treatment and Measurement

Serum and brain BHB levels were quantified using a commercial β-hydroxybutyrate assay kit (Sigma-Aldrich, St. Louis, MO, USA) following the manufacturer’s protocol. Samples were mixed with assay buffer and incubated in the dark at room temperature for 30 min. Absorbance was measured at 450 nm using a SpectraMax M5 microplate reader (Molecular Devices, San Jose, CA, USA). All samples were analyzed in triplicate.

### 4.11. Immunofluorescence Analysis

Animals were anesthetized with sevoflurane and perfused transcardially with saline, followed by 4% paraformaldehyde. Brains were removed, post-fixed overnight in 4% PFA at 4 °C, and cryoprotected in 30% sucrose. Coronal sections (40 μm) were cut using a freezing microtome (Leica CM1950, Leica Biosystems Nussloch GmbH, Nußloch, Germany). For immunohistochemistry, sections were washed in PBS, blocked with 5% normal goat serum and 0.3% Triton X-100 for 1 h at room temperature, then incubated with primary antibodies overnight at 4 °C. The following primary antibodies were used: rabbit anti-CAMK2A (1:1000, A22611, Abclonal, Wuhan, China), mouse anti-NeuN (1:1000, MAB377, Millipore, Burlington, MA, USA) for mature neurons, mouse anti-GFAP (1:1500, A28062, Abclonal) for astrocytes, and mouse anti-DCX (1:200, sc-271390, Santa Cruz, Dallas, TX, USA) for immature neurons, and mouse anti-NeuN (1:1000, A26951, Abclonal, Wuhan, China) and rabbit anti-TH (1:1000, A25683, Abclonal, Wuhan, China) for neuronal labeling. Antibody specificity was confirmed by omission of the primary antibody (no-primary control) and by the expected cellular and regional staining patterns. Sections were washed in PBS and incubated with species-specific secondary antibodies conjugated to Alexa Fluor 488, 555, or 647 (1:500, Invitrogen, Thermo Fisher Scientific, Waltham, MA, USA) for 1 h at 37 °C. Nuclei were counterstained with DAPI. After additional washes, sections were mounted with anti-fade medium (Solarbio, Beijing, China, S2100). Images were acquired using a confocal microscope (LSM880, Zeiss, Dublin, CA, USA) with identical settings across compared groups.

### 4.12. RNA Interference

Small RNA interference and stable knockdown approaches were employed. For siRNA-mediated knockdown, double-stranded siRNAs targeting *Camk2a* were synthesized by YiXueSheng Biosciences Inc. (Shanghai, China). Cells seeded in six-well plates were subjected to transfection with 20 nM siRNA using Lipofectamine RNAiMax (Invitrogen, Thermo Fisher Scientific, Waltham, MA, USA) according to the manufacturer’s instructions, and harvested 48 h later for downstream analyses.

### 4.13. Dopamine and DOPAC Quantification

Hippocampal tissue samples were homogenized in ice-cold PBS and centrifuged at 12,000× *g* for 10 min at 4 °C. The supernatants were collected for ELISA. Dopamine levels were measured using a commercial ELISA kit (Beyotime, Shanghai, China, S0580S) following the manufacturer’s instructions. DOPAC (3,4-dihydroxyphenylacetic acid) levels were determined using a mouse-specific ELISA kit (Shanghai Enzyme-linked Biotechnology, Shanghai, China, SP14936) according to the provided protocol. Absorbance was read at 450 nm using a microplate reader (Thermo Fisher Scientific, Waltham, MA, USA). Sample concentrations were calculated from standard curves and normalized to the total protein content determined by the BCA assay.

### 4.14. Quantification and Statistical Analysis

All cellular and molecular experiments were independently repeated at least three times. Behavioral experiments were performed with 8–10 mice per group, and histological/immunohistochemical analyses used 4 mice per group. Data are presented as the mean ± standard deviation (SD). For comparisons between two groups, an unpaired two-tailed Student’s *t*-test was applied, and the t value and degrees of freedom (df) are reported together with the *p* value. For comparisons involving three or more groups, one-way analysis of variance (ANOVA) was used, followed by Tukey’s post hoc test when variances were equal (Brown–Forsythe test) or Dunnett’s T3 test when unequal. For ANOVA, the F value and the associated degrees of freedom (between groups, within groups) are reported. For metabolomic and proteomic data, differential expression was determined using Student’s *t*-test with thresholds of |log_2_(fold change)| > 1 and *p* < 0.05, and enrichment analysis was performed using Fisher’s exact test. All statistical analyses were performed using GraphPad Prism (version 8.0.2; GraphPad Inc., San Diego, CA, USA). A *p* value < 0.05 was considered statistically significant. All statistical details (t, F, df, and exact *p* values) are provided in Section 2 and corresponding figure legends.

## 5. Conclusions

Our results revealed a hippocampal signaling pathway through which the ketogenic diet enhanced reward learning. We showed that the key ketone body β-hydroxybutyrate (BHB) upregulated neuronal CAMK2A expression, thus activating dopaminergic synaptic signaling. This cascade enhances synaptic plasticity and promotes reward-related behavior. Critically, in vivo knockdown of hippocampal *Camk2a* abolished the pro-reward effects of the diet without affecting systemic ketosis. These findings established the BHB–CAMK2A–dopaminergic signaling axis as a crucial mechanistic link between metabolic state and cognitive function, providing novel insights into how dietary interventions modulate complex brain processes.

## Data Availability

The original contributions presented in this study are included in the article/Supplementary Material. Further inquiries can be directed to the corresponding author.

## Supplementary Materials

The following supporting information can be downloaded at: https://www.mdpi.com/article/10.3390/ijms27083587/s1.

## Abbreviations

The following abbreviations are used in this manuscript:

KD	Ketogenic diet
ND	Normal diet
BHB	β-Hydroxybutyrate
VTA	Ventral tegmental area
NAc	Nucleus accumbens
SPF	Specific pathogen-free
SPT	Sucrose preference test
CPP	Conditioned place preference
OFT	Open field test
HE	Hematoxylin and Eosin
PCA	Principal component analysis
DDC	Dopa-decarboxylase
DEP	Differentially expressed protein
COG	Clusters of Orthologous Groups
GO	Gene Ontology
LTP	Long-term potentiation
TH	Tyrosine hydroxylase
DOPAC	3,4-Dihydroxyphenylacetic acid
3-MT	3-Methoxytyramine

## References

[1] Jeong H., Taylor A., Floeder J.R., Lohmann M., Mihalas S., Wu B., Zhou M., Burke D.A., Namboodiri V.M.K. (2022). Mesolimbic dopamine release conveys causal associations. Science.

[2] Masset P., Tano P., Kim H.R., Malik A.N., Pouget A., Uchida N. (2025). Multi-timescale reinforcement learning in the brain. Nature.

[3] Morales M., Margolis E.B. (2017). Ventral tegmental area: Cellular heterogeneity, connectivity and behaviour. Nat. Rev. Neurosci..

[4] Corkrum M., Covelo A., Lines J., Bellocchio L., Pisansky M., Loke K., Quintana R., Rothwell P.E., Lujan R., Marsicano G. (2020). Dopamine-Evoked Synaptic Regulation in the Nucleus Accumbens Requires Astrocyte Activity. Neuron.

[5] Biane J.S., Ladow M.A., Stefanini F., Boddu S.P., Fan A., Hassan S., Dundar N., Apodaca-Montano D.L., Zhou L.Z., Fayner V. (2023). Neural dynamics underlying associative learning in the dorsal and ventral hippocampus. Nat. Neurosci..

[6] Le Merre P., Esmaeili V., Charrière E., Galan K., Salin P.A., Petersen C.C.H., Crochet S. (2018). Reward-Based Learning Drives Rapid Sensory Signals in Medial Prefrontal Cortex and Dorsal Hippocampus Necessary for Goal-Directed Behavior. Neuron.

[7] Doron A., Rubin A., Benmelech-Chovav A., Benaim N., Carmi T., Refaeli R., Novick N., Kreisel T., Ziv Y., Goshen I. (2022). Hippocampal astrocytes encode reward location. Nature.

[8] Lodge D.J., Grace A.A. (2011). Hippocampal dysregulation of dopamine system function and the pathophysiology of schizophrenia. Trends Pharmacol. Sci..

[9] Farsi Z., Nicolella A., Simmons S.K., Aryal S., Shepard N., Brenner K., Lin S., Herzog L., Moran S.P., Stalnaker K.J. (2023). Brain-region-specific changes in neurons and glia and dysregulation of dopamine signaling in Grin2a mutant mice. Neuron.

[10] Dyńka D., Kowalcze K., Paziewska A. (2022). The Role of Ketogenic Diet in the Treatment of Neurological Diseases. Nutrients.

[11] Yancy W.S., Olsen M.K., Guyton J.R., Bakst R.P., Westman E.C. (2004). A low-carbohydrate, ketogenic diet versus a low-fat diet to treat obesity and hyperlipidemia: A randomized, controlled trial. Ann. Intern. Med..

[12] Wells J., Swaminathan A., Paseka J., Hanson C. (2020). Efficacy and Safety of a Ketogenic Diet in Children and Adolescents with Refractory Epilepsy-A Review. Nutrients.

[13] Qiao Y.N., Li L., Hu S.H., Yang Y.X., Ma Z.Z., Huang L., An Y.P., Yuan Y.Y., Lin Y., Xu W. (2024). Ketogenic diet-produced β-hydroxybutyric acid accumulates brain GABA and increases GABA/glutamate ratio to inhibit epilepsy. Cell Discov..

[14] van der Louw E., van den Hurk D., Neal E., Leiendecker B., Fitzsimmon G., Dority L., Thompson L., Marchió M., Dudzińska M., Dressler A. (2016). Ketogenic diet guidelines for infants with refractory epilepsy. Eur. J. Paediatr. Neurol..

[15] Xu Y., Jiang C., Wu J., Liu P., Deng X., Zhang Y., Peng B., Zhu Y. (2022). Ketogenic diet ameliorates cognitive impairment and neuroinflammation in a mouse model of Alzheimer’s disease. CNS Neurosci. Ther..

[16] Nagpal R., Neth B.J., Wang S., Craft S., Yadav H. (2019). Modified Mediterranean-ketogenic diet modulates gut microbiome and short-chain fatty acids in association with Alzheimer’s disease markers in subjects with mild cognitive impairment. EBioMedicine.

[17] Zhou Z., Kim K., Ramsey J.J., Rutkowsky J.M. (2023). Ketogenic diets initiated in late mid-life improved measures of spatial memory in male mice. GeroScience.

[18] Wang X.Y., Zhang X.G., Sang Y.J., Chong D.Y., Sheng X.Q., Wang H.Q., Yang C.F., Yan G., Sun H.X., Li C.J. (2022). The neonatal ketone body is important for primordial follicle pool formation and regulates ovarian ageing in mice. Life Metab..

[19] Ang Q.Y., Alexander M., Newman J.C., Tian Y., Cai J., Upadhyay V., Turnbaugh J.A., Verdin E., Hall K.D., Leibel R.L. (2020). Ketogenic Diets Alter the Gut Microbiome Resulting in Decreased Intestinal Th17 Cells. Cell.

[20] Moya-Garzon M.D., Wang M., Li V.L., Lyu X., Wei W., Tung A.S., Raun S.H., Zhao M., Coassolo L., Islam H. (2025). A β-hydroxybutyrate shunt pathway generates anti-obesity ketone metabolites. Cell.

[21] Shippy D.C., Wilhelm C., Viharkumar P.A., Raife T.J., Ulland T.K. (2020). β-Hydroxybutyrate inhibits inflammasome activation to attenuate Alzheimer’s disease pathology. J. Neuroinflamm..

[22] Newman J.C., Verdin E. (2017). β-Hydroxybutyrate: A Signaling Metabolite. Annu. Rev. Nutr..

[23] Shimazu T., Hirschey M.D., Newman J., He W., Shirakawa K., Le Moan N., Grueter C.A., Lim H., Saunders L.R., Stevens R.D. (2013). Suppression of oxidative stress by β-hydroxybutyrate, an endogenous histone deacetylase inhibitor. Science.

[24] Lan Z., Chen A., Li L., Ye Y., Liang Q., Dong Q., Wang S., Fu M., Li Y., Liu X. (2022). Downregulation of HDAC9 by the ketone metabolite β-hydroxybutyrate suppresses vascular calcification. J. Pathol..

[25] Weng X., Pan L., Ma X., Luo W., Su H., Pei Z., Dong Z., Liu L., Yang J., Gao P. (2025). Ketogenic diet and β-hydroxybutyrate inhibit HDAC1 to preserve vascular smooth muscle cell function in thoracic aortic aneurysm. J. Adv. Res..

[26] Won Y.J., Lu V.B., Puhl H.L., Ikeda S.R. (2013). β-Hydroxybutyrate modulates N-type calcium channels in rat sympathetic neurons by acting as an agonist for the G-protein-coupled receptor FFA3. J. Neurosci..

[27] Rigter P.M.F., Wallaard I., Aghadavoud Jolfaei M., Kingma J., Post L., Elgersma M., Elgersma Y., van Woerden G.M. (2022). Adult Camk2a gene reinstatement restores the learning and plasticity deficits of Camk2a knockout mice. iScience.

[28] He Y., Ren Y., Chen X., Wang Y., Yu H., Cai J., Wang P., Ren Y., Xie P. (2025). Neural and molecular investigation into the paraventricular thalamus for chronic restraint stress induced depressive-like behaviors. J. Adv. Res..

[29] Xiao K., Li Y., Chitwood R.A., Magee J.C. (2023). A critical role for CaMKII in behavioral timescale synaptic plasticity in hippocampal CA1 pyramidal neurons. Sci. Adv..

[30] Liu H., Yang H., Fang Y., Li K., Tian L., Liu X., Zhang W., Tan Y., Lai W., Bian L. (2020). Neurotoxicity and biomarkers of zinc oxide nanoparticles in main functional brain regions and dopaminergic neurons. Sci. Total Environ..

[31] Dedic N., Kühne C., Jakovcevski M., Hartmann J., Genewsky A.J., Gomes K.S., Anderzhanova E., Pöhlmann M.L., Chang S., Kolarz A. (2018). Chronic CRH depletion from GABAergic, long-range projection neurons in the extended amygdala reduces dopamine release and increases anxiety. Nat. Neurosci..

[32] Bueno N.B., de Melo I.S., de Oliveira S.L., da Rocha Ataide T. (2013). Very-low-carbohydrate ketogenic diet v. low-fat diet for long-term weight loss: A meta-analysis of randomised controlled trials. Br. J. Nutr..

[33] Pepino M.Y., Eisenstein S.A., Bischoff A.N., Klein S., Moerlein S.M., Perlmutter J.S., Black K.J., Hershey T. (2016). Sweet Dopamine: Sucrose Preferences Relate Differentially to Striatal D2 Receptor Binding and Age in Obesity. Diabetes.

[34] Liu B., Qiao L., Liu K., Liu J., Piccinni-Ash T.J., Chen Z.F. (2022). Molecular and neural basis of pleasant touch sensation. Science.

[35] Yi L.X., Tan E.K., Zhou Z.D. (2024). Tyrosine Hydroxylase Inhibitors and Dopamine Receptor Agonists Combination Therapy for Parkinson’s Disease. Int. J. Mol. Sci..

[36] Haavik J. (1997). L-DOPA is a substrate for tyrosine hydroxylase. J. Neurochem..

[37] Wang Y., Tong Q., Ma S.R., Zhao Z.X., Pan L.B., Cong L., Han P., Peng R., Yu H., Lin Y. (2021). Oral berberine improves brain dopa/dopamine levels to ameliorate Parkinson’s disease by regulating gut microbiota. Signal Transduct. Target. Ther..

[38] Salling M.C., Faccidomo S.P., Li C., Psilos K., Galunas C., Spanos M., Agoglia A.E., Kash T.L., Hodge C.W. (2016). Moderate Alcohol Drinking and the Amygdala Proteome: Identification and Validation of Calcium/Calmodulin Dependent Kinase II and AMPA Receptor Activity as Novel Molecular Mechanisms of the Positive Reinforcing Effects of Alcohol. Biol. Psychiatry.

[39] Neal E.G., Chaffe H., Schwartz R.H., Lawson M.S., Edwards N., Fitzsimmons G., Whitney A., Cross J.H. (2008). The ketogenic diet for the treatment of childhood epilepsy: A randomised controlled trial. Lancet Neurol..

[40] Newman J.C., Covarrubias A.J., Zhao M., Yu X., Gut P., Ng C.P., Huang Y., Haldar S., Verdin E. (2017). Ketogenic Diet Reduces Midlife Mortality and Improves Memory in Aging Mice. Cell Metab..

[41] Tosefsky K.N., Zhu J., Wang Y.N., Lam J.S.T., Cammalleri A., Appel-Cresswell S. (2024). The Role of Diet in Parkinson’s Disease. J. Park. Dis..

[42] Akita T., Aoto K., Kato M., Shiina M., Mutoh H., Nakashima M., Kuki I., Okazaki S., Magara S., Shiihara T. (2018). De novo variants in CAMK2A and CAMK2B cause neurodevelopmental disorders. Ann. Clin. Transl. Neurol..

[43] Sosa M., Plitt M.H., Giocomo L.M. (2025). A flexible hippocampal population code for experience relative to reward. Nat. Neurosci..

[44] Sosa M., Plitt M.H., Giocomo L.M. (2024). Hippocampal sequences span experience relative to rewards. bioRxiv.

