# Supplementary material for: Ketogenic Diet Promotes Reward Learning by Upregulating Hippocampal CAMK2A Expression and Activating Dopamine Synaptic Signaling
Source: Int J Mol Sci. 2026 Apr 17;27(8):3587. doi: 10.3390/ijms27083587 (PMC13116303; doi:10.3390/ijms27083587)
Supplement: Supplementary file 1 [file ijms-27-03587-s001.zip › ijms-4231942-supplementary.pdf]

Table S1.

Reagent	Resource	Identifier
**Antibodies**		
Anti-CAMK2A	Abclonal	Cat# A22611
anti-NeuN	Millipore	Cat# MAB377
anti-GFAP	Abclonal	Cat# A28062
anti-DCX	Santa Cruz	Cat# sc271390
anti-GluA1	Cell Signaling Technology	Cat# 13185
Anti-P-GluA1	Cell Signaling Technology	Cat# 8084
Anti-Actin	Abclonal	Cat# AC026
**Chemicals, Peptides, and Recombinant Proteins**		
GCaMP6s	Addgene	Cat# 100844
3-Hydroxybutyric acid sodium salt	Sigma-Aldrich	Cat# 298360
Trichostatin A (TSA)	Selleck	Cat# S1045
Romidepsin (FK228)	Selleck	Cat# S3020
**Primers**		
Primer	Sequence	
Camk2a-F	GCTCTTCGAGGAATTGGGCAA	
Camk2a-R	CCTCTGAGATGCTGTCATGTAGT	
Gnaq-F	AAGGTGTCTGCTTTTGAGAATCC	
Gnaq-R	CGTCGTCTATCATAGCATTCCTG	
Prkca-F	ATGTCACAGTACGAGATGCAAAA	
Prkca-R	GCTTTCATTCTTGGGATCAGGAA	
Gria3-F	TCCGGGCGGTCTTCTTTTTAG	
Gria3-R	TCCACCTATGCTGATGGTGTT	
Gng2-F	ATGAGCGTATGGAGAAATCGTTT	
Gng2-R	GGAGATTGCCTACCAAGGACAA	
Grin2a-F	GACCCCAAGAGCCTCATCAC	
Grin2a-R	CTGGATGGACGCTCCAAACT	

Actb-F	CATGTACGTTGCTATCCAGGC	
Actb-R	CTCCTTAATGTCACGCACGAT	
si*Camk2a*-F	AAAGUCAUGCAAAGAAAACAG	
si*Camk2a*-R	GUUUUCUUUGCAUGACUUUAU	
**Software and Algorithms**		
Graphpad Prism 10.0	Graphpad Software	https://www.graphpad.com
Image J	NIH	https://imagej.nih.gov/ij/
